# Editorial: Learning, Perception, and Collaboration for Robots in Industrial Environments

**DOI:** 10.3389/frobt.2022.888971

**Published:** 2022-04-14

**Authors:** Jacopo Aleotti, Matteo Saveriano, Riccardo Monica

**Affiliations:** ^1^ Robotics and Intelligent Machines Laboratory, Department of Engineering and Architecture, University of Parma, Parma, Italy; ^2^ Automatic Control Laboratory, Department of Industrial Engineering, University of Trento, Trento, Italy

**Keywords:** human-robot collaboration, deep learning methods, robot perception, industrial robots, motion and task planning

Automation and robotics in real operative conditions, like modern factories, are constantly evolving. Collaborative robots represent one of the major changes in industrial robotics and their market is undergoing strong growth. A collaborative robot is designed for direct interaction with a human to improve the work experience and to reduce the risk of injuries. Among the most important challenges in the development of collaborative robots are those related to performing tasks in unstructured environments, moving in a shared workspace, manipulating objects, and learning from expert operators (Caccavale et al., 2019). In order to achieve such goals, collaborative robots can take advantage of recent innovations in machine learning, as well as of advances in algorithms for robot perception and new sensor technologies (Chiaravalli et al., 2020; Monica and Aleotti, 2020).

The goal of this Research Topic was to investigate new methods for intelligent collaborative robots. Authors were encouraged to submit papers discussing new research findings that have been successfully evaluated by conducting experiments in real environments. A particular focus of the Research Topic has been the development of novel methods and solutions for task planning, human-robot interaction and programming by demonstration.

The Research Topic is a collection of four original research papers. In the work by Iturrate et al. a complete system for learning gluing and dispensing tasks from a single demonstration was presented that encompasses a full pipeline of demonstration, encoding and execution. The main contribution is the design of a novel unified controller for kinesthetic teaching and execution of in-contact tasks.

In their work, Mangin et al. present a method to plan supportive actions for a robot in collaborative human-robot assembly tasks. The method models uncertainty in the task by means of a Partially Observable Markov Decision Process. Unlike most works in literature, where the goal is to model uncertainty in the physical interactions or the environment, the goal here is to handle uncertainty in the human’s intentions. The resulting system supports on-the-fly replanning and error recovery.

The coordination of a task, shared between a human and a robot, is investigated in the work by Angleraud et al. from a high-level perspective. The presented coordination system is based on a set of high-level actions triggered by human commands. Both a speech-based and a graphical interface are developed and tested in typical industrial tasks like hand-over and kitting.

The work by Kramberger et al. presents a comprehensive framework for the human-robot cooperative assembly of timber structures, comprising the design of novel interlocking joints, Learning from Demonstration strategies for cooperative assembly under different operating conditions, and an enhanced simulation of the process through a digital twin.
